# Gastric Medullary Carcinoma with Sporadic Mismatch Repair Deficiency and a* TP53* R273C Mutation: An Unusual Case with Wild-Type* BRAF*

**DOI:** 10.1155/2017/3427343

**Published:** 2017-08-03

**Authors:** Brett M. Lowenthal, Theresa W. Chan, John A. Thorson, Kaitlyn J. Kelly, Thomas J. Savides, Mark A. Valasek

**Affiliations:** ^1^Department of Pathology, University of California, San Diego, 200 West Arbor Drive, MC 8320, San Diego, CA 92103, USA; ^2^Department of Surgery, University of California, San Diego, 200 West Arbor Drive, MC 8320, San Diego, CA 92103, USA; ^3^Department of Medicine, Division of Gastroenterology, University of California, San Diego, 200 West Arbor Drive, MC 8320, San Diego, CA 92103, USA

## Abstract

Medullary carcinoma has long been recognized as a subtype of colorectal cancer associated with microsatellite instability and Lynch syndrome. Gastric medullary carcinoma is a very rare neoplasm. We report a 67-year-old male who presented with a solitary gastric mass. Total gastrectomy revealed a well-demarcated, poorly differentiated carcinoma with an organoid growth pattern, pushing borders, and abundant peritumoral lymphocytic response. The prior cytology was cellular with immunohistochemical panel consistent with upper gastrointestinal/pancreaticobiliary origin. Overall, the histopathologic findings were consistent with gastric medullary carcinoma. A mismatch repair panel revealed a mismatch repair protein deficient tumor with loss of MLH1 and PMS2 expression. BRAF V600E immunostain (VE1) and* BRAF* molecular testing were negative, indicating a wild-type gene. Tumor sequencing of* MLH1* demonstrated a wild-type gene, while our molecular panel identified* TP53* c.817C>T (p.R273C) mutation. These findings were compatible with a sporadic tumor. Given that morphologically identical medullary tumors often occur in Lynch syndrome, it is possible that mismatch repair loss is an early event in sporadic tumors with p53 mutation being a late event. Despite having wild-type* BRAF*, this tumor is sporadic and unrelated to Lynch syndrome. This case report demonstrates that coordinate ancillary studies are needed to resolve sporadic versus hereditary rare tumors.

## 1. Introduction

The World Health Organization (WHO) Digestive System has known medullary carcinoma to be a subtype of colorectal carcinoma and linked with microsatellite instability and Lynch syndrome. The 4th edition of the WHO has added “gastric medullary carcinoma,” which is also known as “lymphoepithelioma-like carcinoma” and “carcinoma with lymphoid stroma,” as a distinct diagnostic entity [1]. Gastric medullary carcinoma is an extremely rare gastric neoplasm typically presenting as early stage disease and in elderly males [2, 3]. Despite the synonymous determination by the WHO, gastric medullary carcinoma is frequently associated with microsatellite instability-high (MSI-H), while lymphoepithelioma-like carcinoma is associated with Epstein Barr Virus (EBV) infection [4–6]. Gastric medullary carcinoma has been defined histologically as comprising more than 90% of poorly differentiated solid type adenocarcinoma with associated lymphocytic infiltrate, demonstrating pushing growth pattern at the tumor edge, and excluding other special types of gastric carcinoma [7].

Here we present a case of a 67-year-old male with a pT2 N0 mismatch repair protein deficient,* MLH1* wild-type gastric medullary carcinoma with a* TP53* c.817C>T (p.R273C) mutation by molecular testing. Overall, the clinical, morphologic, and molecular findings were consistent with a sporadic tumor unrelated to Lynch syndrome.

## 2. Case Presentation

**Figure 1:**
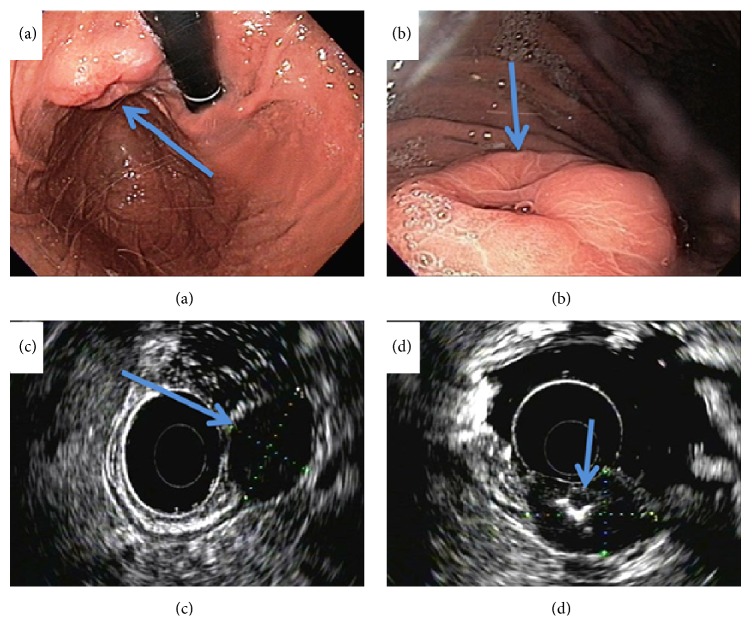
Esophagogastroduodenoscopy (EGD) and endoscopic ultrasound (EUS) of the gastric mass. (a)-(b) EGD shows the 3.5 × 2.5 × 1.5 cm subepithelial gastric mass with central umbilication (blue arrow). (c)-(d) EUS shows the gastric mass is hypoechoic (blue arrow).

A 67-year-old Filipino male with a past medical history of hypertension presented to his outside gastroenterologist for a routine screening colonoscopy that was significant for diverticulosis. He complained of upper abdominal pain and bloating to his gastroenterologist. He then underwent an esophagogastroduodenoscopy (EGD), which demonstrated a gastric cardia/fundus mass. The patient was then referred to our institution for additional work-up. An EGD with endoscopic ultrasound was performed at our institution, which redemonstrated the 3.5 cm subepithelial hypoechoic gastric mass that appeared to arise from the gastric muscular wall with central umbilication and ulceration (Figures 1(a)–1(d)). The initial differential diagnosis included gastrointestinal stromal tumor versus a gastric leiomyoma. The remaining stomach, duodenum, and esophagus were unremarkable. A fine needle aspiration showed groups and single enlarged atypical cells with anisonucleosis, pleomorphism, and prominent nucleoli consistent with adenocarcinoma. The malignant cells were positive for CK7, Villin, and CDX2 immunostains and negative for CK20, CD117, and CD56 immunostains. This immunoprofile and this cytomorphology were consistent with an adenocarcinoma of upper gastrointestinal or pancreatobiliary primary origin.

**Figure 2:**
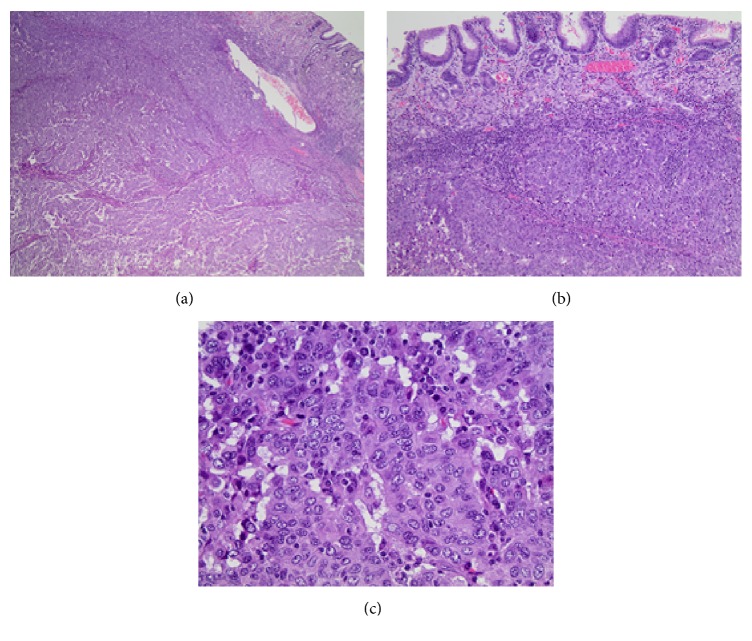
Histologic H&E stained total gastrectomy. (a) Well-demarcated, poorly differentiated carcinoma with an organoid growth pattern and pushing borders ((a) 20x; (b) 100x). (c) High power view of organoid growth pattern and abundant peritumoral lymphocytic response (400x).

A positron emission tomography/computed tomography (PET/CT) scan from skull to midthigh was significant for increased hypermetabolic activity in the gastric cardia/fundus mass, which was consistent with malignancy and no evidence of hypermetabolic metastases or lymph node involvement. Given this presumed early stage tumor and proximal location, the surgical team decided to proceed with a total gastrectomy. The gastric resection specimen contained a well-circumscribed, poorly differentiated carcinoma with an organoid growth pattern and pushing borders centered on the muscularis propria (Figures 2(a)-2(b)). There was an abundant peritumoral lymphocytic response (Figure 2(c)). Although the mass focally extended into the surface gastric mucosa, there was no discernible precursor lesion such as intraepithelial neoplasia or dysplasia. Taken together with the morphologic features and the immunoprofile performed on the prior cytologic fine needle aspiration, the tumor was best categorized as a gastric medullary carcinoma. The surgical resection margins and all seventeen lymph nodes were negative for carcinoma. The background gastroesophageal mucosa demonstrated goblet cell metaplasia without dysplasia. The pathological staging was a pT2 N0.

**Figure 3:**
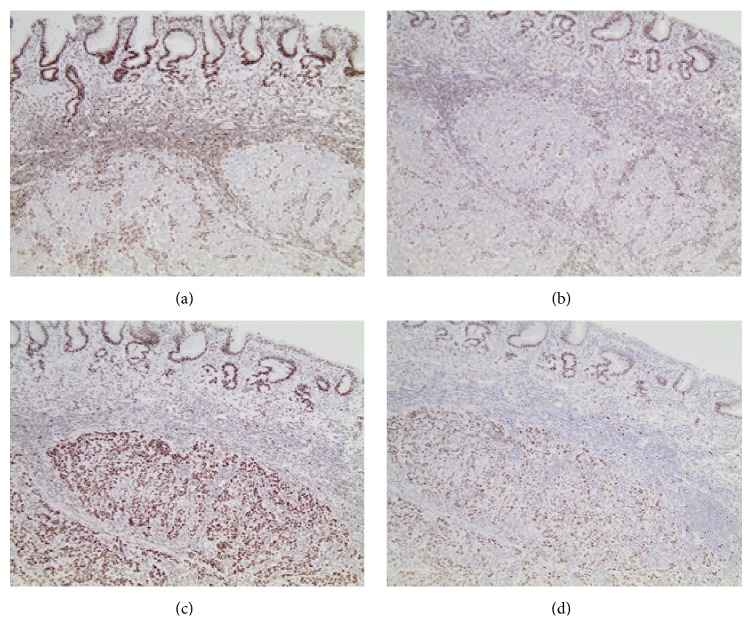
Mismatch repair protein expression immunohistochemical profile (100x). The gastric medullary carcinoma is located at the bottom half of each field, while the overlying unremarkable gastric mucosa is located at the top portion of each field. (a) Loss of MLH1 expression. (b) Loss of PMS2 expression. (c) Intact MSH2 expression. (d) Intact MSH6 expression.

The Ki-67 proliferation index was greater than 80%. In situ hybridization for EBV was negative. A mismatch repair panel was performed by immunohistochemical stains. The invasive tumor cells demonstrated loss of normal MLH1 (Figure 3(a)) and PMS2 (Figure 3(b)) protein expression, with retention of normal MSH2 (Figure 3(c)) and MSH6 (Figure 3(d)) protein expression. BRAF V600E immunohistochemical stain (VE1) and* BRAF* molecular testing were negative, indicating a wild-type gene. Next generation sequencing of DNA from the tumor demonstrated a wild-type* MLH1* gene. Our standard molecular sequencing panel was performed on the tumor tissue and additionally identified a* TP53* c.817C>T (p.R273C) mutation. The* TP53* gene mutation was not present in the patient's germline, as evidenced by sequencing of DNA from a peripheral blood sample. With the patient's advanced age and these molecular findings, the tumor was compatible with a sporadic mutation and not a germline mutation.

The patient was discharged with no surgical complications on postoperative day 7. He did not undergo chemotherapy or radiation. Thirty-five months since the diagnosis of gastric medullary carcinoma, the patient was followed in surgery clinic. The EGD and imaging studies revealed no recurrent or metastatic disease.

## 3. Discussion

Gastric medullary carcinoma, a new WHO diagnostic entity, is a very rare subtype of gastric cancer that is histologically characterized by a sharply demarcated, poorly differentiated carcinoma with organoid/syncytial growth pattern with pushing borders and an associated lymphocytic inflammatory response [1, 7]. It is typically seen in elderly men, associated with MSI-H, presents at an early clinical and pathologic stage, and has an improved prognosis compared to gastric cancers of other special types [6].

Our patient presented with a pathologic stage T2 N0 gastric medullary carcinoma with a high proliferative rate and negative EBV by in situ hybridization. The invasive tumor cells demonstrate loss of normal MLH1 and PMS2 protein expression, with retention of normal MSH2 and MSH6 protein expression. These results indicated the loss of normal DNA mismatch repair function within the tumor. This loss of protein expression may be associated with the presence of a germline, or heritable, mutation in one of the mismatch repair genes associated with Lynch syndrome [8]. The loss may also be associated with sporadic hypermethylation of the MLH1 promoter region. To help differentiate between sporadic and heritable gastrointestinal syndromes, such as Lynch, BRAF V600E, and* MLH1* hypermethylation testing must be performed. The BRAF V600E mutation by immunohistochemistry and molecular sequencing was negative. This indicated a wild-type gene expression of the patient's gastric medullary carcinoma. We also confirmed that the patient had wild-type* MLH1* by gene sequencing.

Our institution performed a standard molecular sequencing panel of 47 genes on this tumor, which revealed a* TP53* c.817C>T (p.R273C) gene mutation. Since medullary carcinomas are associated with MSI-H, a consideration should always be given to Lynch syndrome in an appropriate clinical context. With regard to the patient's age of 67 years at diagnosis, lack of other clinical or familial findings supportive for a genetic syndrome, and the molecular results, this case was compatible with a sporadic mutation and not germline. There are three underlying molecular pathways in gastric carcinomas, which include p53 mutation, E-cadherin mutation, and hypermethylation in the promotor region [1]. One study demonstrated that there were no differences in p53 or E-cadherin mutation expression when comparing medullary and nonmedullary gastric carcinomas but did show that MSI-H was identified significantly more often in medullary versus nonmedullary gastric carcinomas [6]. Given that medullary carcinomas often occur in Lynch syndrome as MSI-H, it is possible in this case that mismatch repair loss was an early event for sporadic tumors with* TP53* mutation being a late genetic hit event.

In summary, we presented a very rare case of gastric medullary carcinoma with sporadic mismatch repair deficiency for MLH1 and PMS2, wild-type* BRAF* gene, and a* TP53* c.817C>T (p.R273C) gene mutation. This case report validates the importance of ancillary immunohistochemical and molecular studies to differentiate sporadic and hereditary rare tumors, such as gastric medullary carcinoma.
